# Classification of wheat diseases using deep learning networks with field and glasshouse images

**DOI:** 10.1111/ppa.13684

**Published:** 2023-01-10

**Authors:** Megan Long, Matthew Hartley, Richard J. Morris, James K. M. Brown

**Affiliations:** ^1^ Department of Crop Genetics John Innes Centre Norwich UK; ^2^ Department of Computational and Systems Biology John Innes Centre Norwich UK; ^3^ Present address: European Molecular Biology Laboratory European Bioinformatics Institute Hinxton UK

**Keywords:** brown rust, convolutional neural network (CNN), deep learning, septoria, wheat, yellow rust

## Abstract

Crop diseases can cause major yield losses, so the ability to detect and identify them in their early stages is important for disease control. Deep learning methods have shown promise in classifying multiple diseases; however, many studies do not use datasets that represent real field conditions, necessitating either further image processing or reducing their applicability. In this paper, we present a dataset of wheat images taken in real growth situations, including both field and glasshouse conditions, with five categories: healthy plants and four foliar diseases, yellow rust, brown rust, powdery mildew and Septoria leaf blotch. This dataset was used to train a deep learning model. The resulting model, named CerealConv, reached a 97.05% classification accuracy. When tested against trained pathologists on a subset of images from the larger dataset, the model delivered an accuracy score 2% higher than the best‐performing pathologist. Image masks were used to show that the model was using the correct information to drive its classifications. These results show that deep learning networks are a viable tool for disease detection and classification in the field, and disease quantification is a logical next step.

## Graphical abstract

A convolutional neural network, CerealConv, was trained to classify wheat diseases from images with 97% classification accuracy. When compared with trained pathologists, CerealConv produces comparable performance on test images.

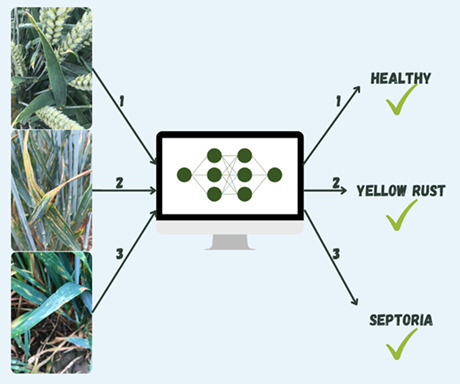

## 1 INTRODUCTION

Wheat is a staple crop in which the control of multiple diseases by breeding resistant varieties, applying pesticides, or other measures is important for maintaining yield and quality. Two of the most commercially important wheat diseases in the UK and many other countries are yellow rust, also called stripe rust, caused by the basidiomycete fungus *Puccinia striiformis* f. sp. *tritici* (Pst) (Liu & Hambleton, 2010), and Septoria tritici blotch, also known as Septoria leaf blotch or simply Septoria, caused by the ascomycete fungus *Zymoseptoria tritici* (formerly *Mycosphaerella graminicola*) (Hardwick et al., 2001). Distinguishing these two diseases is sometimes problematic because of their similar appearance at certain stages in their life cycle (Brown, 2021). The symptoms that are most commonly associated with these diseases are clearly different, but they are not representative of the full array of possible symptoms over the course of infection. At their most recognizable stages, yellow rust is easily diagnosed by the stripes of orange/yellow uredinial pustules that form on the leaves of wheat plants, whereas mature Septoria appears on the leaf as necrotic yellow‐to‐brown lesions restricted by the veins of the leaf with many small black pycnidia (Agriculture and Horticulture Development Board [AHDB], 2020). In most of Europe, the uredinial stage of yellow rust is formed a few weeks earlier than the pycnidial stage of Septoria. Confusion can arise, however, at two stages. In the early stages of each disease, yellow rust and Septoria both appear as elongated areas of chlorosis. Later on, as yellow rust gets to the later stages of its life cycle, the distinctive orange spores fall from the leaf leaving necrotic lesions with black telia (AHDB, 2020), which can be confused with mature Septoria lesions that are formed at approximately the same time (Schirrmann et al., 2021). This presents problems even for experienced pathologists when they have limited time to look closely at each plot and can thus generate mistakes in the assessment of wheat varieties and fungicides. The new genotypes of Pst, which have infected wheat in northern Europe since 2011, produce much more abundant telia than the only clone that was present until 2010 (Rodriguez‐Algaba et al., 2020), making it more difficult to discriminate late yellow rust and mature Septoria.

To further complicate the problem, another important wheat disease, brown or leaf rust, caused by *Puccinia triticina* (Bolton et al., 2008; Goyeau et al., 2006), produces orange/brown pustules on the leaves of wheat plants. These brown rust pustules can be difficult to distinguish from the earliest stages of yellow rust and immature pycnidia of Septoria. The final foliar disease relevant to this paper is powdery mildew, caused by *Blumeria graminis*, which is an important disease in many parts of the world (Dubin & Duveiller, 2011). Whilst several other foliar diseases affect wheat across the world, the four mentioned here are considered the most important in the UK (AHDB, 2020).

In order to control disease on any crop, it is important to be able to identify the disease present. Often, this requires specialist pathology knowledge, which is not always readily available to farmers or agronomists. An automated system for disease detection would allow any user to identify diseases on their crop easily, without the need for specialist skills, and allow them to implement a cost‐effective fungicide programme. Advances in machine learning, specifically deep learning, have enabled game‐changing advances in several applications including text recognition (Jaderberg et al., 2015; Lecun et al., 1998) and image recognition (He et al., 2016; Simonyan & Zisserman, 2015) amongst others.

Deep learning methods have shown great promise in the detection of diseases on various crops using images as input. One notable dataset used in multiple studies is the Plant Village dataset (Hughes & Salathe, 2016), which contains over 50,000 images of diseased leaves taken in controlled conditions with simple backgrounds such as a single colour. Multiple studies have used the whole or part of this dataset (Amara et al., 2017; Brahimi et al., 2017; Ferentinos, 2018; Mohanty et al., 2016; Rangarajan et al., 2018; Saleem et al., 2020; Zhang et al., 2018). Other studies have used their own images, also collected in controlled conditions (Liu et al., 2018). Although other studies have collected their own, more complex images under field conditions, these study datasets have often contained few classes; DeChant et al. (2017) gained accuracies of 96.7%, but it is unclear how well their method would perform with multiple classes. Conversely, some studies have used multiple classes with few images per category (Singh et al., 2020). In these cases, it is unlikely that any network would perform well in the field as the training data would not be comprehensive enough to cover the range of realistic conditions. Some studies have used data augmentation, whereby an image is digitally flipped, rotated, or otherwise transformed, thus increasing the number of images for training the network. However, this does not add any new information or variation to the dataset.

In the work reported here, we aimed to test if deep learning networks are capable of handling complex images taken in realistic growth situations for multicategory classification of wheat diseases. We developed and trained a convolutional neural network (CNN), which we have named CerealConv, for the identification and classification of diseased wheat leaf images taken in field and glasshouse conditions. For the five predefined categories of Septoria, yellow rust, brown rust, mildew and healthy, our network achieved a classification accuracy of over 97%. When tested on a smaller dataset against manual classification by five pathologist participants, our network performed with an accuracy of 2% higher than the most accurate pathologist. Given that the images included diverse irrelevant information in the background in addition to the affected (or healthy) leaves, we tested whether or not CerealConv uses the correct information to drive disease classification. Our work suggests that deep learning methods can handle real field condition images well and can perform at least as well as expert wheat pathologists.

## 2 MATERIALS AND METHODS

### 2.1 Dataset determination

We collected images of infected and healthy wheat from various locations across the UK and Ireland in the summer of 2019. Photographs were taken using various iOS and Android smartphones and a digital camera. Prior to preprocessing, the resolution of the images ranged from 6 to 16 megapixels, depending on the capture device. Diseased (or healthy) leaves were photographed whilst still attached to the plants (both seedling and adult), with up to five leaves being the focus of each image. These leaves were photographed both above and below the canopy, at a range of distances from the plant, from approximately 20 cm to 1 m, and with a range of backgrounds including plants within the field plot, other vegetation, soil and sky. We captured images from multiple angles to represent the variation that would occur with different people using the model in the future. For the disease categories, only plants with visible symptoms were photographed, including the earliest visible symptoms to assist in early detection.

Field locations included farmers' fields and plant breeding trial plots. The majority of field photographs were taken in plant breeders' trials containing thousands of wheat lines. A minority were taken in farmers' fields containing diverse commercial varieties. Photographs were taken over a 3‐week period in varying weather conditions. Many mildew images were taken in a glasshouse containing diverse lines from genetic and plant breeding experiments. Each photography location was identified by a pathologist as having only one disease present and the resulting photographs were all labelled with that disease. At least four different locations were used to photograph each category. We took care to include so many different conditions firstly, so the model could work in all situations and secondly, to reduce the risk of the model learning features that were not related to the disease.

Every photograph was checked visually to assess the quality, both of the photograph and the information contained. Any photographs that were out of focus or showed no important information (because it contained no relevant part of any plant for example) were removed. In cases where the photograph possibly had more than one disease present or if the image did not match the disease label from the site, these were also removed. Photographs with fingers or boots in the foreground or background were left in the dataset unless they were obstructing the main information. All images that passed quality control were assigned a label corresponding to the disease present. Within the dataset, each of the five categories was divided into a train set (60%), a validation set (20%) and a test set (20%).

To evaluate CerealConv against expert pathologists, we created a smaller dataset of 999 images using images from the test dataset. In this dataset, we included all images that the trained network failed to classify correctly and an even sampling of all categories and prediction scores. This ensured that the dataset contained a selection of images that, from the network's perspective, had varying degrees of difficulty in classification. The images were shuffled randomly to ensure that there was no way to predict which disease would appear next. A tagging system was created that loaded each image onto the screen for a user to classify. Five expert crop pathologists, with differing backgrounds and levels of experience, took part in the experiment. Each user saw the images in the same order and was asked to classify them individually to obtain a range of results representing the breadth of knowledge expected to be found in breeding companies. The tags 1–5 were used to represent each of the five categories contained within the dataset. Once a tag was assigned, the next image was automatically loaded. The system collected the allocated tags for each participant.

As an additional validation of CerealConv's performance, a separate experiment was conducted to test if the CNN was reliably using the correct information to classify images into the five categories. This was done by placing black rectangles over diseased leaves (or healthy leaves in the foreground for the “healthy” category) that we considered would be most informative for classification. When CerealConv used the correct information for classification, a large reduction in accuracy was observed for the partially masked images compared to the originals.

### 2.2 Training deep learning networks

**FIGURE 1:**
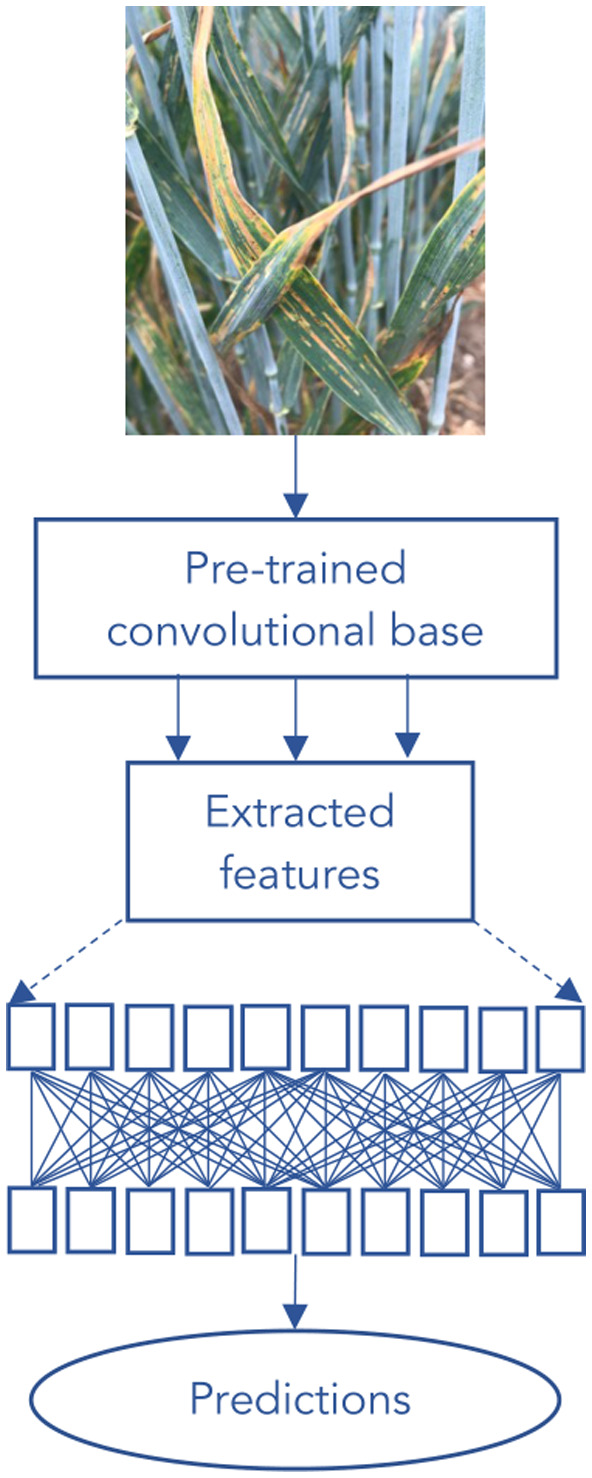
Flow chart of the transfer learning process. Transfer learning takes the knowledge learned whilst training on a larger dataset and applies it to the new dataset (in this case our wheat images). We removed the part of the pretrained network that provides classifications and sent our images once through the part of the network that extracts features, the pretrained convolutional base. These features were then used for training a short classifier network, which learnt to classify the images from our dataset using the extracted features.

We first tested the following pretrained networks: VGG16 (Simonyan & Zisserman, 2015), Inception V3 (Szegedy et al., 2016), Mobilenet (Howard et al., 2017), Xception (Chollet, 2017). All of these networks have been trained on the ImageNet dataset (Deng et al., 2009), which contains 1.2 million images classified into 1000 categories. We used transfer learning with the pretrained ImageNet parameters for each of these networks to gain classifications of our own wheat images (Figure 1). We removed the part of the pretrained network that provides classifications and sent our images once through the part of the network that extracts features, the pretrained convolutional base. These features were then used for training a short classifier network, which learnt to classify the images from our dataset using the extracted features. The classifier network was the same for each pretrained network. Training for this was carried out using a RMSProp optimizer (Hinton & Tieleman, 2012).

**FIGURE 2:**
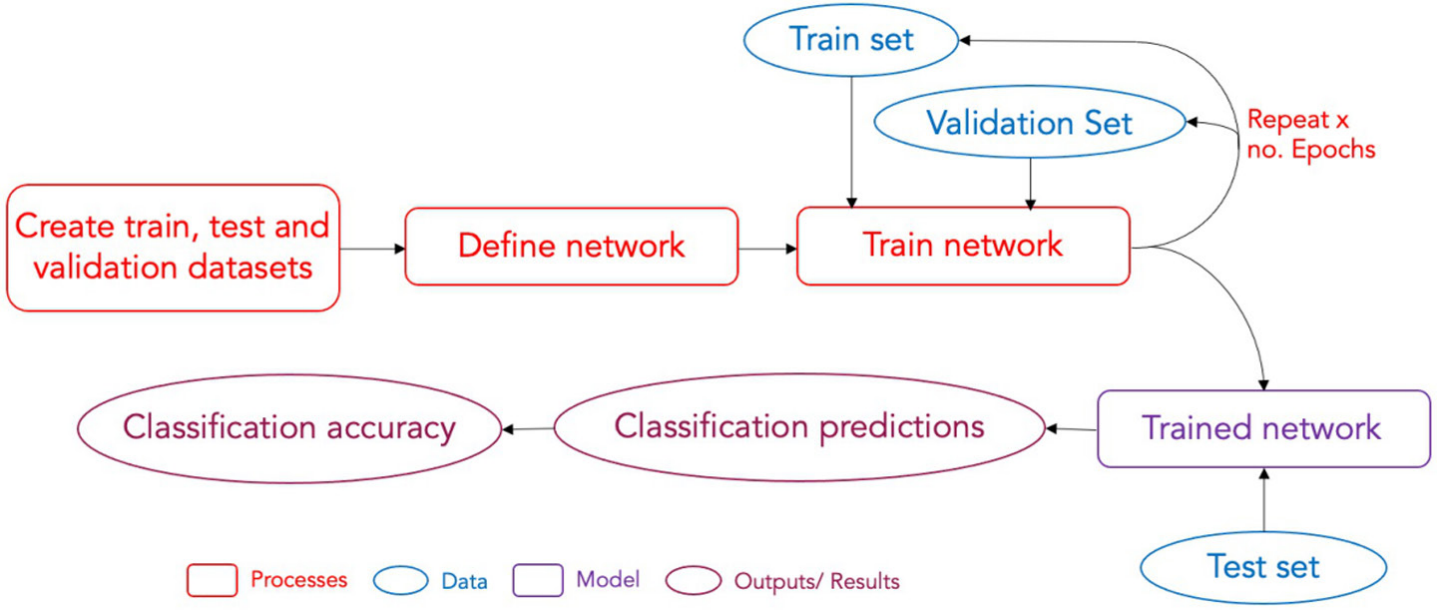
Flow chart of the deep learning process. First, the dataset is split into three smaller sets. For this work, 60% of the images were put into the train set, and 20% into each of the validation and test sets. The design of the network (network architecture) was then defined ready for training. The train set was fed through the network in small batches for a set number of epochs, allowing the network to learn features from the images for classification predictions. The internal parameters of the model were adjusted to maximize the accuracy of its classifications. The validation set was used to monitor the performance after each epoch on an independent dataset to reduce over‐fitting (indicated by a divergence of prediction accuracy between the train set and validation dataset). Using the validation set in this way, as part of an optimization procedure, can introduce a dependence on the validation set. Therefore, once the network had been trained for a set number of epochs, or once the classification accuracy had converged to a stable level, the network performance was evaluated against a different dataset, the test set. The network provided predicted classifications for the images in the test set, to gauge the accuracy of the network when presented with never‐before‐seen data.

Alongside the pretrained networks, we developed and trained our own CNN models, with the aim of testing if greater accuracy could be achieved by a network designed and trained specifically to classify plant diseases. Several network designs were explored and evaluated. The CNNs were developed using keras v. 2.2.0 (Chollet, 2015) in Python v. 3.5.1. Training was carried out using a RMSProp optimizer (Figure 2).

The training and validation set were used to determine the best hyperparameters, for example, the learning rate, number of epochs for training and batch size. Each model was then retrained using both the training and validation set, and evaluated against the test set. All values are reported in Table S1. To evaluate the performance, the classification accuracy was found by calculating the percentage of classifications that the model got correct. We also found the F1 score (Goutte & Gaussier, 2005), which is used as a measure of performance for classification models. We calculated a macro‐averaged F1 score for each model by first using Equation (1) for each category, and then calculating the average of those results.

(1) $$F1=2\left(\text{precision}\times \text{recall}\right)/\left(\text{precision}+\text{recall}\right)$$

All networks were trained using the Norwich Bioscience Institute's high‐performance computing (HPC) facilities. The pretrained networks used central processing units (CPUs) on the HPC clusters, whilst our own networks were trained using graphics processing units (GPUs) to decrease training time.

## 3 RESULTS

### 3.1 Collection and curation of wheat disease images under realistic growth conditions

To evaluate the potential for automated disease detection in the field, we collected images that reflected conditions found in typical field situations. Over 19,000 images of wheat leaves with either Septoria, yellow rust, brown rust, mildew, or no disease were collected from various locations across the UK and Ireland in the summer of 2019 and these formed the basis of our dataset.

**FIGURE 3:**
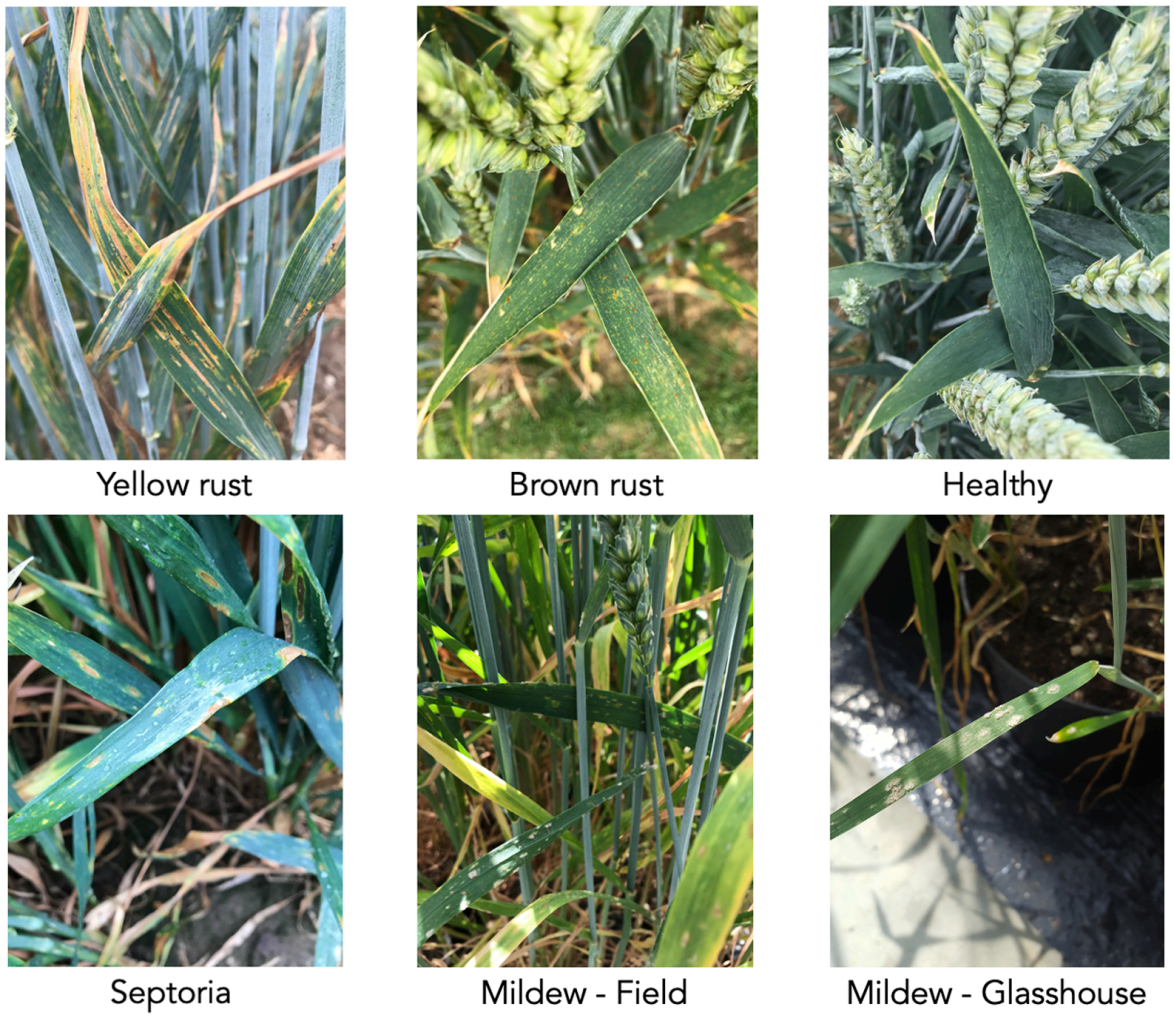
Example images from each wheat disease category in the dataset, taken in field conditions: Top row—yellow rust (left), brown rust (middle), healthy (no disease) (right). Bottom row—Septoria tritici blotch (left), mildew in the field (middle), mildew in glasshouse (right).

Example images from the dataset are shown in Figure 3. Table 1 summarizes the number of images contained within each category for the full dataset, the smaller dataset for the pathologist tagging experiment, and the masked image experiment.

**TABLE 1:** The number of images of each category of healthy and diseased wheat that passed quality control.

Category	Number of images		
	Full dataset	Pathologist tagging experiment	Masked images
Brown rust	2502	128	148
Healthy	2274	122	99
Mildew	2942	161	97
Septoria	7054	349	175
Yellow rust	4388	239	118
Total	19,160	999	637

### 3.2 Testing and comparing the performance of different deep learning models

We experimented with four pretrained networks (MobileNet, InceptionV3, VGG16 and Xception). For each network, we gained the best results by resizing the input images to four times their original size. Once the features had been extracted, they were used to train a short classifier network consisting of one fully connected layer with 256 neurons, dropout (where units and their connections were randomly dropped from the neural network; Srivastava et al., 2014) and the prediction output layer. It was trained with a learning rate of 1 × 10^−4^ and a batch size of 128. See Table S1 for input image sizes and the number of training epochs for each network.

**FIGURE 4:**
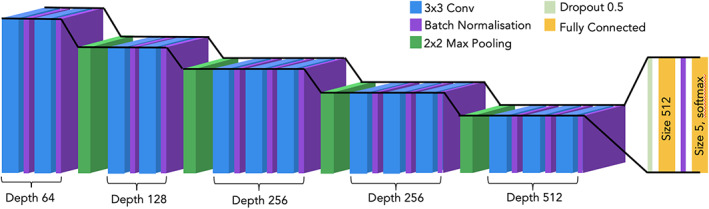
The architecture of our CerealConv model used in these experiments. The blue blocks depict convolutional layers, the purple batch normalization, the dark green max pooling, the light green dropout and yellow fully connected layers. Information about the layer hyperparameters (depth, filter size, dropout value) is also included.

**FIGURE 5:**
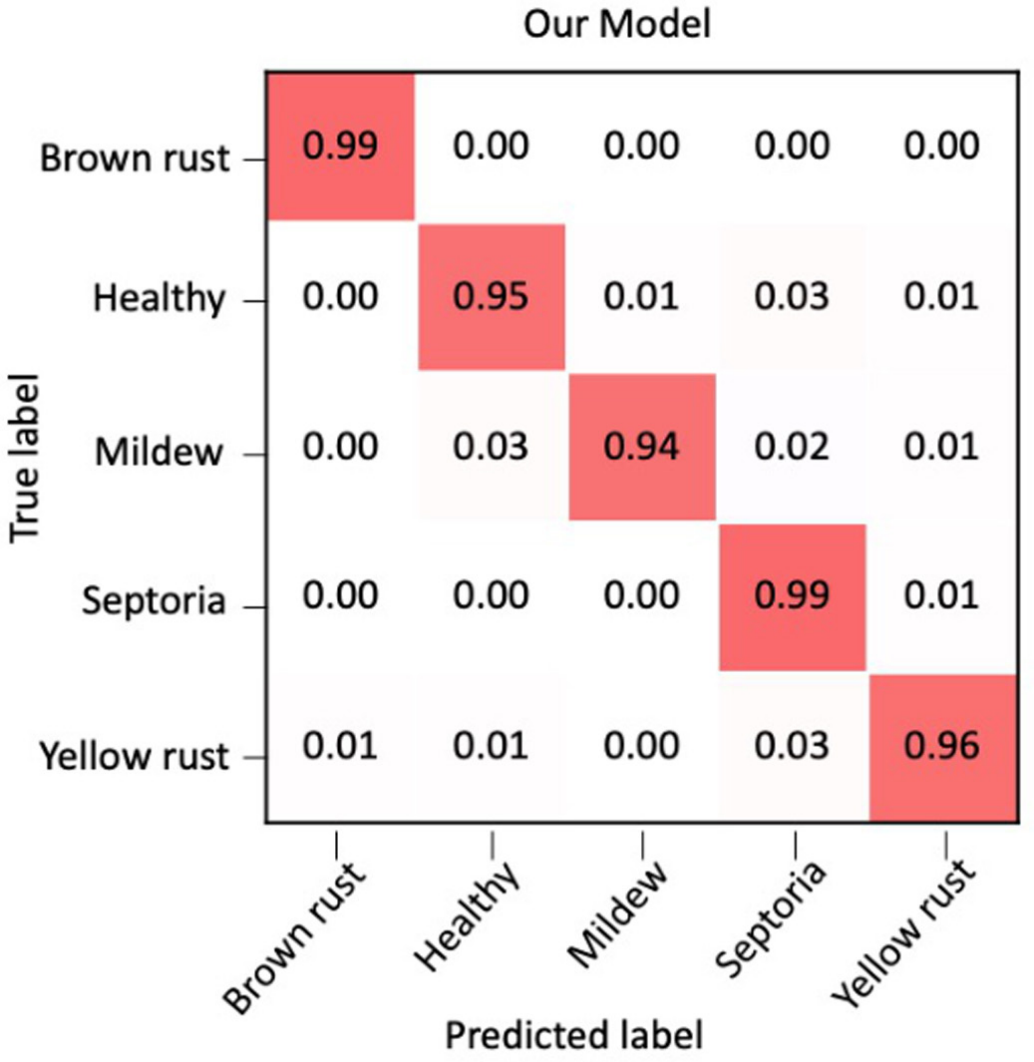
Confusion matrix, summarizing the prediction results of the convolutional neural network (CNN) model, CerealConv, and showing the proportion of correct and incorrect predictions for each wheat disease category.

The success of transfer learning suggested that deep learning networks can deal with complex images such as those taken under real field conditions for detecting wheat diseases. For use in the field, however, higher accuracies would be most beneficial. Therefore, we explored other networks to optimize the classification accuracy. We performed experiments with multiple network architectures to find the number and combination of layers that worked best for this problem. We also experimented with different input image sizes, batch sizes, learning rates and number of training epochs used. Our best‐performing model was a CNN (LeCun et al., 1999), consisting of 13 convolutional layers, with batch normalization (Ioffe & Szegedy, 2015), max pooling (where only the maximum value of the area being convolved is used) and dropout (Figure 4). During preprocessing, the images were resized to 256 × 256 pixels. No data augmentation methods were used. This model, which we have named CerealConv, was trained for 75 epochs, with a batch size of 8 and a learning rate of 1 × 10^−4^. See Figure S1 for training and validation accuracy and loss plots. The confusion matrix of the CNN model CerealConv's classifications shows that CerealConv performs consistently well over all the categories with only minimal confusion between categories (Figure 5, Table 2).

**TABLE 2:** Comparison of accuracy of wheat disease classification by four pretrained networks and our model, CerealConv, using our test dataset.

Model used	Classification accuracy on test dataset (%)	F1 score^a^
MobileNet	91.46	0.90
InceptionV3	91.41	0.91
VGG16	85.16	0.83
Xception	89.87	0.89
CerealConv	97.05	0.97

^a^ F1 score is a measure of performance for classification models and is calculated as F1 = 2(precision × recall)/(precision + recall) (Goutte & Gaussier, 2005).

### 3.3 Evaluating the performance of CerealConv against expert pathologists

The dataset used in this experiment contained 999 images representing samples of varying levels of difficulty in classification (based on the network's performance). This dataset included 111 images that the network had classified incorrectly and 888 images that were correctly classified by the network. CerealConv's prediction accuracy for this dataset was thus 88.9%, which was higher than any individual person and significantly higher than the mean of the human scorers, 85.9% (contingency table test, χ^2^ = 6.68, 1 *df*, *p* = 0.01). The pathologists did not vary significantly in their accuracy (χ^2^ = 4.65, 4 *df*, *p* = 0.3).

**FIGURE 6:**
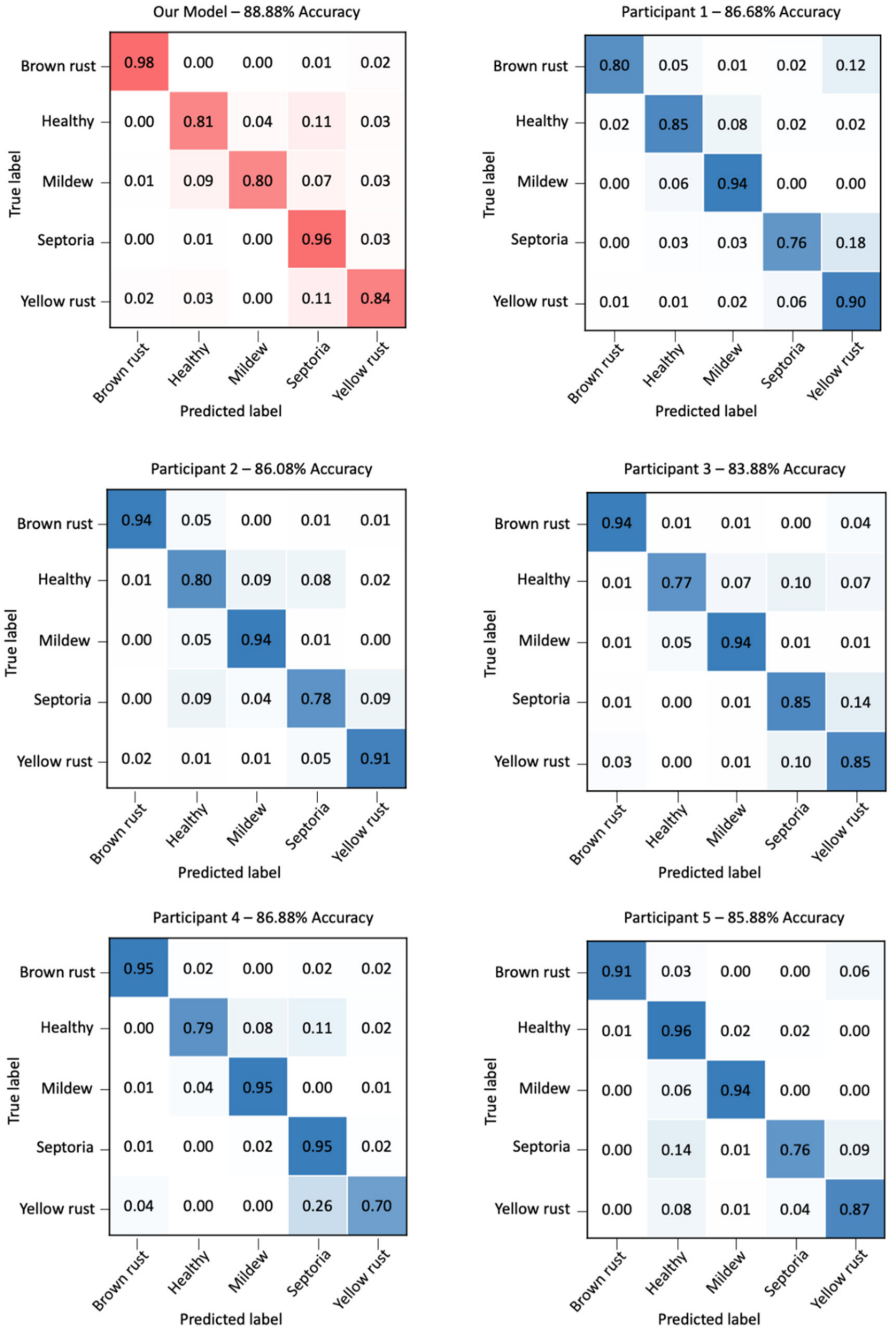
Confusion matrices of the classification of wheat disease by the CerealConv model and five pathologists, using the smaller dataset of 999 images. The proportion of correct and incorrect predictions for each category shows that our model achieved an overall accuracy on this dataset of 88.88%, 2% higher than the most accurate pathologist.

The human participants for this experiment were five expert crop pathologists, from four different companies or institutions, each with substantial experience in identifying and scoring these diseases (Table 3). The confusion matrices in Figure 6 show the results of the trained network compared with the results of the five pathologist participants. Our network classified each category with an accuracy of 80% or higher, with mildew being the category with the most incorrect classifications. In contrast to this, each of the five participants classified mildew with 94%–95% accuracy, making it one of the highest accuracy classes. Of the 999 images, 643 images were correctly classified by all five participants.

**TABLE 3:** Years of experience and specialization of each of the five participant plant pathologists.

Participant	Years of experience	Specialization
1	35	Cereal diseases, in particular mildew, yellow rust and Septoria, especially in field trials
2	40+	Cereal pathologist for a major breeding company
3	20	Wheat disease observation plots, mainly for QTL mapping work
4	10	Scoring trial plots for wheat disease, in particular rusts and Septoria
5	12	Common European cereal diseases

Another difference between the results of the network and the pathologists was the classification of the Septoria category. Here, the network performed extremely well, gaining 96% accuracy, but all but one of the pathologists were less accurate, with a range of 76%–85%. For these four pathologists, the main source of incorrect classification was yellow rust, with the healthy category also being significantly misclassified by two of the four. The pathologist who performed similarly to the network on the Septoria category instead showed a dip in classification accuracy in the yellow rust category. Here, almost all the misclassifications were Septoria.

Overall, CerealConv classified the 999 images faster (approximately 1 h on a Mac desktop) than the pathologists, who took close to 3 hours for the same dataset.

### 3.4 Use of masked images to ensure CerealConv is using the correct information to drive classification

**FIGURE 7:**
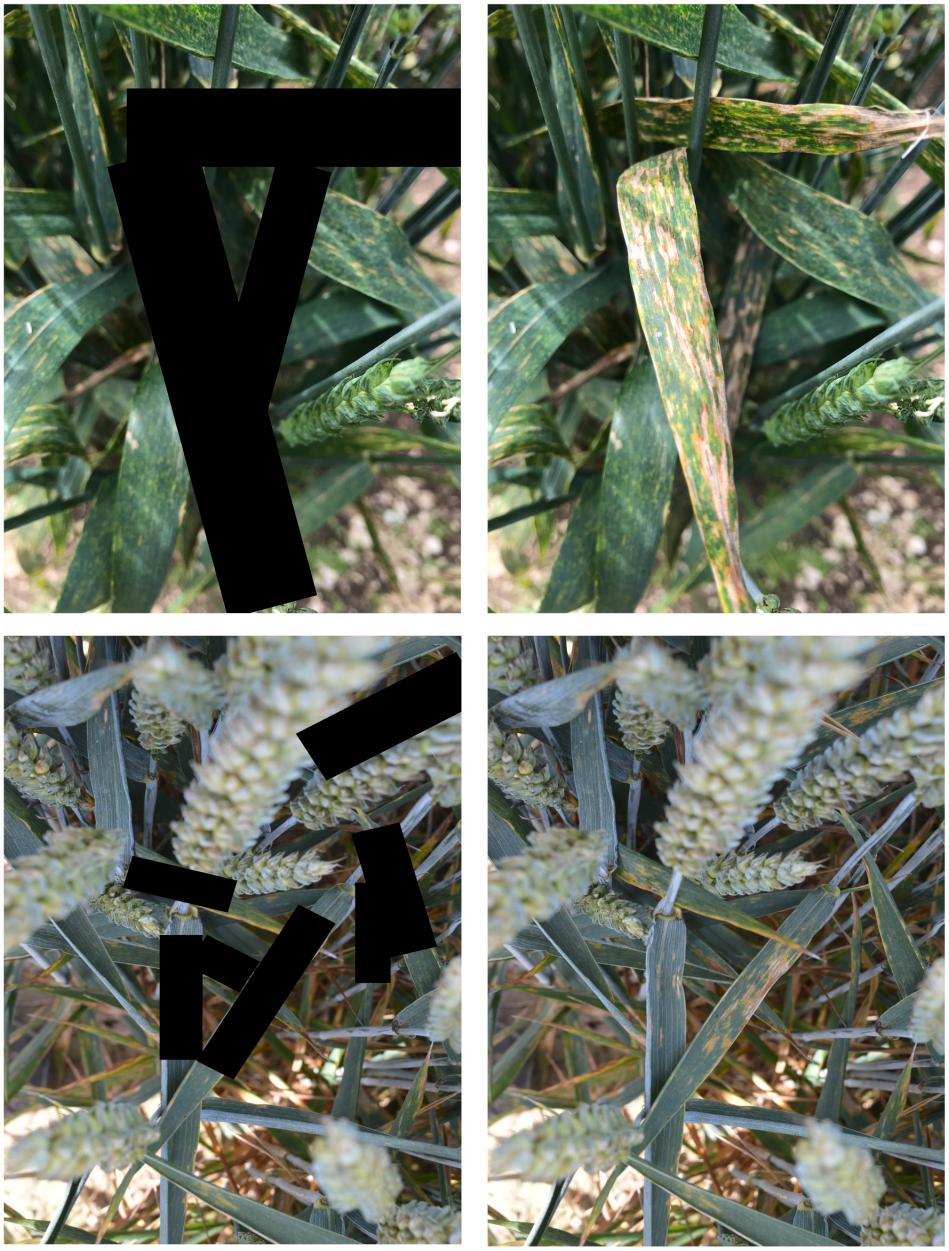
Examples of masked (left) and unmasked images of diseased wheat used to determine the drivers of classification by the CerealConv model. Black masks were added to cover the diseased parts of the image.

Given the high accuracy of CerealConv, we wanted to determine whether it was using the correct parts of the images (the disease lesions and the leaves) to make its classifications. We theorized that with the mask covering the leaf and disease information in the image, a deep learning model would have trouble correctly classifying the images so would “guess” rather than make an informed decision; in this case, we would expect a drop in classification accuracy for the masked image dataset. Examples of the masked images used in this experiment are shown in Figure 7.

**FIGURE 8:**
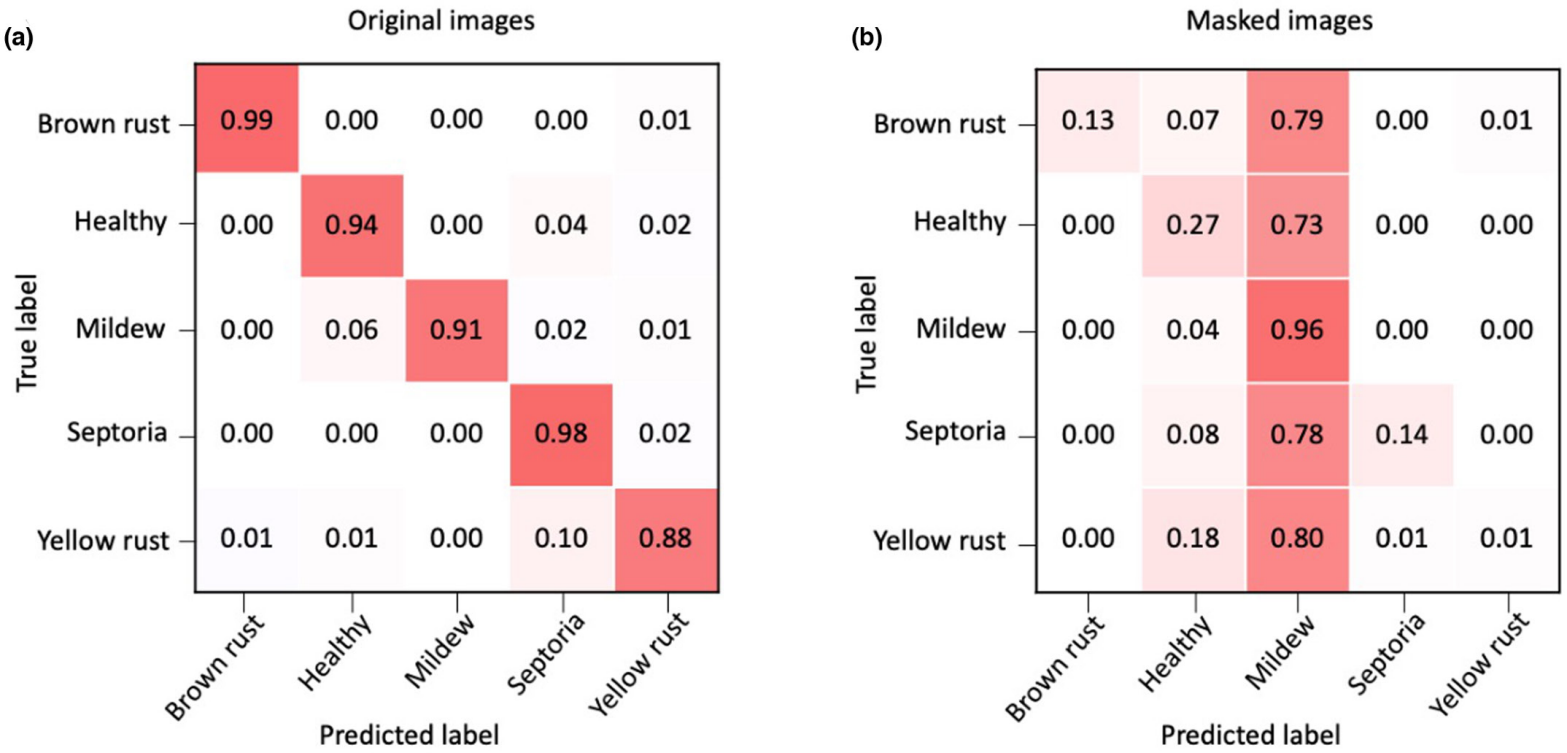
Confusion matrices of the classifications of wheat disease by the CerealConv model when presented with the original images (a) and the same images with masks (b). The masked images have significantly poorer classification across all classes other than mildew.

The classification results for the original versions of the images used in this experiment and the masked images are shown in the confusion matrices in Figure 8. There is a clear difference between the classifications of the two sets of images. As expected, the confusion matrix for the original images echoes that of the full dataset, where the network classifies the images with high accuracy, making only a small number of misclassifications.

With the masked images, there was a clear bias towards mildew classification over all the categories. This means that there was only a small difference between the mildew category classifications over both image sets, implying that the model may have used background information to classify mildew images. There was also a smaller tendency to incorrect classification as healthy; this was not as obvious as the bias towards mildew, but it was the only other category that was consistently picked as an incorrect classification over all categories.

Figure 8 shows that, other than the mildew category, CerealConv performs much better when classifying the original images than the masked ones. This indicates that CerealConv performs as expected using the leaf information for making a classification decision.

## 4 DISCUSSION

This study aimed to evaluate the viability of deep learning methods, specifically CNNs, for identifying wheat diseases from images taken in realistic growth conditions. Several studies preprocessed their images to remove complex background information (Barbedo, 2018) or did not include background information in their images at all (Liu et al., 2018; Mohanty et al., 2016). In recent years, datasets acquired in the field have become more widely used (Li et al., 2021), but many datasets have contained few images per category, meaning they could not fully represent the range of conditions found in the field. Our dataset includes images that capture the complex conditions found typically in the field and represent typical examples of the kind of images that automated disease detection in realistic situations would need to be able to deal with.

In many cases, the datasets collected and used for plant disease detection with deep learning, such as the ImageNet dataset (Liu & Wang, 2021), are still significantly smaller than those used for other purposes. Our dataset aims to overcome this bottleneck for wheat disease, whilst also being as comprehensive as possible to ensure that it can perform well in the field. Importantly, our dataset does not include any data for presymptomatic infections. Whilst this would be valuable for the early detection of disease, it was not viable for this study, which was based on visible symptoms. Capturing images of symptomless plants would probably have required specialist equipment, such as hyperspectral cameras, and image analysis techniques outside the visible spectrum, which was beyond the scope of our study.

Furthermore, we did not include images with multiple infections in our dataset. At this stage, we aimed to prove that deep learning models could classify each disease with a high accuracy individually given a large, comprehensive dataset of complex field images. Work to identify multiple diseases simultaneously would be a useful development in the future.

It would now be beneficial to test the CerealConv model in the field. Considering the comprehensive and realistic training data, it is reasonable to expect that it would perform relatively well for single diseases. This would allow a farmer to check the disease on his or her plants and implement the correct fungicide application, without necessarily involving a trained pathologist. The performance would naturally drop when encountering infections from multiple diseases.

Although CerealConv classified the 999 images faster than the pathologists, the speed of the model's computation could be increased further by using GPUs and parallelization. This would be easier to achieve than recruiting more trained pathologists to perform the same job.

An important thing to note is that this classification was performed on static images. The deep learning network achieved an impressive accuracy for such data, at least comparable to that of expert crop pathologists, but it is likely that the performance of the pathologists would increase with real plants. In a real field situation, a pathologist would be able to look closer, change their viewing angle, and obtain information that would be available in the field but not from static images. Thus, it is probable that the accuracy of pathologists would be higher in field situations.

Furthermore, we should consider the discrepancies between the ability of different humans to classify. For example, in this experiment, there were 160 cases where only one participant gave an incorrect classification. When shown the images again as a group, in many cases there was a unanimous decision on the classification of the image, indicating a human error aspect to this experiment. However, in some cases where two or more participants incorrectly classified an image and were shown it again as a group, there was still difficulty in deciding which disease was present.

Comparison of the classification accuracy between the masked and unmasked images suggested that the network was correctly identifying diseased parts of the plant for the Septoria, yellow rust, brown rust and healthy categories, as masking resulted in a steep drop in prediction accuracy. For the mildew images, the prediction accuracy using masked images remained comparable to that for the original images, suggesting that other, non‐disease‐related features might have been driving the classification. This is supported by our model's higher rate of misclassification of mildew images than the other diseases in the experiment with pathologist participants.

To understand this, the conditions in which the photographs of different diseases were taken should be considered. Almost all the incorrect classifications of the masked images were healthy or mildew. Misclassifications as healthy probably arose from having little disease and much greenery present in the background. The issue with the mildew images may have resulted from them being collected predominantly in glasshouse conditions, rather than in the field. The black pots used in the glasshouse, which are present in many of the mildew images, may have caused many of the masked images to be classified as mildew in the absence of any other disease information, because the black masks may have been mistaken for plant pots.

Further research is required to explain the above observations, which highlight two points. First, it would be important to include the same range of conditions over all categories of the dataset to reduce the risk of the model learning to differentiate categories on the basis of irrelevant signals. For example, if one category were photographed in rain, sun and cloud, then all four other categories should also be photographed in these conditions. Second, it is desirable to develop explainable models for which the pixels driving the classification can be identified and the associated patterns in the images related back to biology. Several approaches are under development that could transform the current black‐box nature of deep learning to make them more accessible and interpretable (Carter et al., 2019; Selvaraju et al., 2020).

The next step would be to use deep learning methods to quantify the amount of disease present and to give a numerical or ordinal score. Whereas a classification model is useful for identifying the presence of a disease on a plot or field, a scoring model would allow pathologists to quantify severity. This would be highly beneficial for the rapid scoring of large numbers of field plots whilst not needing the continuous involvement of a highly skilled pathologist. Such applications, however, are likely to require significant further development before any system will be ready for use in the field.

In conclusion, in this study we evaluated the potential of deep learning approaches for the detection and diagnosis of wheat diseases using real field images. It is important to be able to classify diseases from complex images that give an accurate representation of realistic field conditions, so the results can be used in the field by breeders, farmers and agronomists. The results of our work demonstrate that deep learning models are capable of handling complex images of plant diseases taken in real field conditions, without the need to remove the background information.

The trained network marginally outperformed expert plant pathologists in terms of classification accuracy and, less surprisingly, did so more quickly. Speed is significant as, generally, a pathologist may only have time to go into the field and score a trial once during the field season, whereas a deep learning model could be deployed as a mobile phone application, or on a drone if the network was retrained using aerial images, using photographs taken on multiple occasions by a person with no specialist expertise in pathology. This means that a more accurate picture could be built of the progression of a disease over time. For wheat in northern Europe, the results presented here show the potential for using deep learning to distinguish Septoria leaf blotch from yellow rust, thus increasing the accuracy of variety evaluation and other field trials.

Our case study also highlighted the potential pitfalls of using datasets that are unbalanced in terms of the variability of conditions within each category and the need to carefully validate what is driving the classification. The development of methods that aid the interpretation of network models will help address these issues.

## CONFLICT OF INTEREST

The authors declare that the research was conducted in the absence of any commercial or financial relationships that could be construed as a potential conflict of interest.

## Supporting information


Figure S1

Table S1

## Data Availability

The 999 selected images used in the experiment to test pathology experts are available at https://zenodo.org/record/7573133. The full dataset is available on request from the authors. [Correction added on 02 February 2023, after first online publication: The link to Zenodo is updated in this version].
