# Supplementary figures and images for: Classification of wheat diseases using deep learning networks with field and glasshouse images
Source: Plant Pathol. 2023 Jan 10;72(3):536–47. doi: 10.1111/ppa.13684 (PMC10953319; doi:10.1111/ppa.13684)

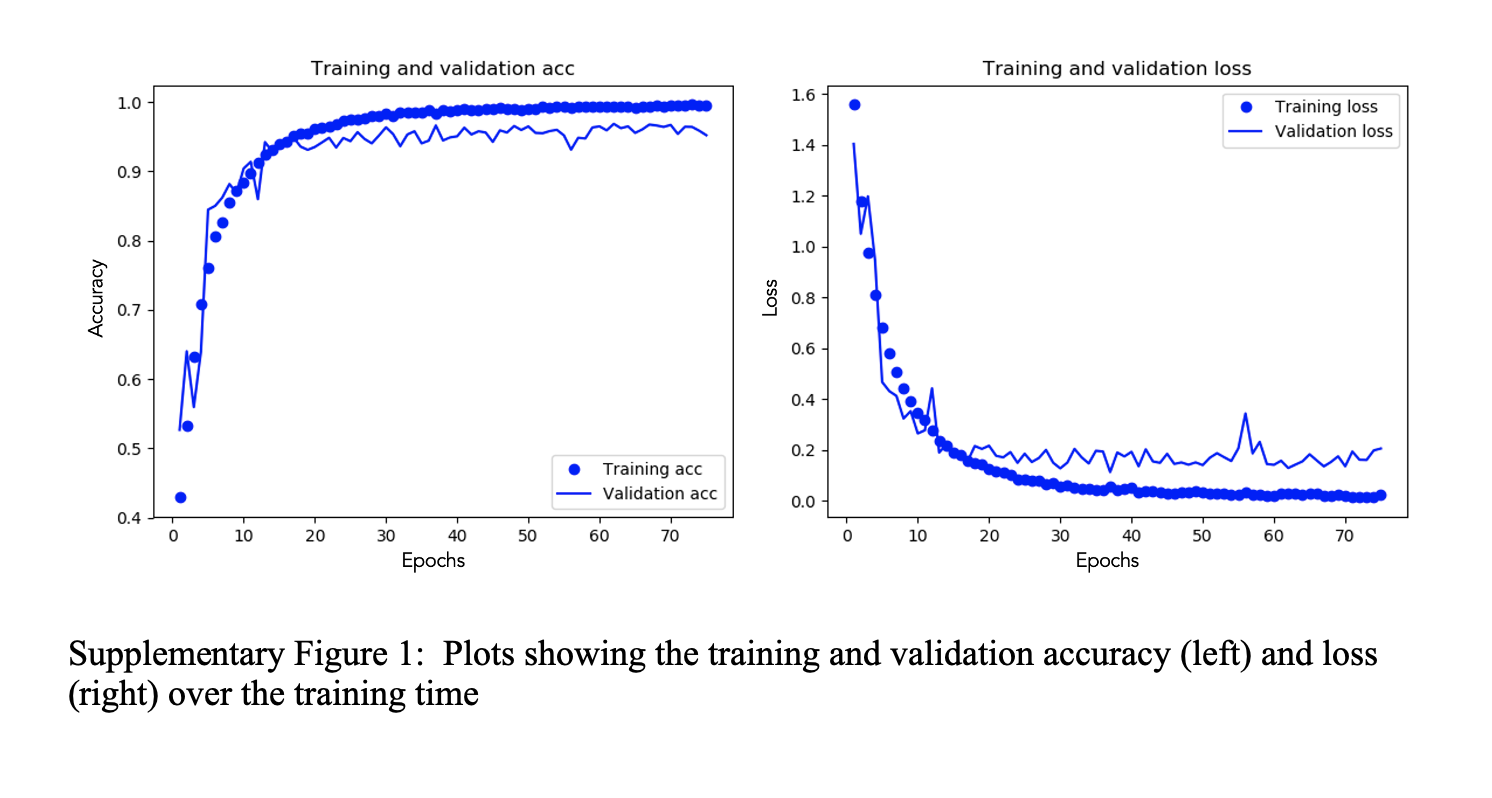

Supplement: Supplementary file 1 — Figure S1 [file PPA-72-536-s001.tiff]
