# Supplementary material for: Classification of wheat diseases using deep learning networks with field and glasshouse images
Source: Plant Pathol. 2023 Jan 10;72(3):536–47. doi: 10.1111/ppa.13684 (PMC10953319; doi:10.1111/ppa.13684)
Supplement: Supplementary file 2 — Table S1 [file PPA-72-536-s002.docx]

| **Models used** | **Input image size for feature extraction (pixels)** | **Number of training epochs** | **Final training accuracy** |
| --- | --- | --- | --- |
| InceptionV3 | 600 x 600 | 20 | 99.46% |
| MobileNet | 532 x 532 | 15 | 99.91% |
| VGG16 | 532 x 532 | 30 | 99.67% |
| Xception | 600 x 600 | 15 | 99.84% |
| CerealConv | 256 x 256 | 75 | 99.55% |

Table S1: Input image sizes and number of training epochs, plus the final training accuracies for each model.
